# Differential gene expression in *Schistosoma japonicum *schistosomula from Wistar rats and BALB/c mice

**DOI:** 10.1186/1756-3305-4-155

**Published:** 2011-08-05

**Authors:** Jinbiao Peng, Hongxiao Han, Geoffrey N Gobert, Yang Hong, Weibin Jiang, Xinzhi Wang, Zhiqiang Fu, Jinming Liu, Yaojun Shi, Jiaojiao Lin

**Affiliations:** 1 Shanghai Veterinary Research Institute, Chinese Academy of Agricultural Sciences, Key Laboratory of Animal Parasitology, Ministry of Agriculture, 518 Ziyue road, Minhang, Shanghai 200241, China; 2 Queensland Institute of Medical Research, Herston, Queensland, Australia; 3 Yangzhou university, College of Veterinary Medicine, Yangzhou, Jiangsu. 225009, China

## Abstract

**Background:**

More than 46 species of mammals can be naturally infected with *Schistosoma japonicum *in the mainland of China. Mice are permissive and may act as the definitive host of the life cycle. In contrast, rats are less susceptible to *S. japonicum *infection, and are considered to provide an unsuitable micro-environment for parasite growth and development. Since little is known of what effects this micro-environment has on the parasite itself, we have in the present study utilised a *S. japonicum *oligonucleotide microarray to compare the gene expression differences of 10-day-old schistosomula maintained in Wistar rats with those maintained in BALB/c mice.

**Results:**

In total 3,468 schistosome genes were found to be differentially expressed, of which the majority (3,335) were down-regulated (≤ 2 fold) and 133 were up-regulated (≥ 2 fold) in schistosomula from Wistar rats compared with those from BALB/c mice. Gene ontology (GO) analysis revealed that of the differentially expressed genes with already established functions or close homology to well characterized genes in another organisms, many are related to important biological functions or molecular processes. Among the genes that were down-regulated in schistosomula from Wistar rats, some were associated with metabolism, signal transduction and development. Of these genes related to metabolic processes, areas including translation, protein and amino acid phosphorylation, proteolysis, oxidoreductase activities, catalytic activities and hydrolase activities, were represented. KEGG (Kyoto Encyclopedia of Genes and Genomes) pathway analysis of differential expressed genes indicated that of the 328 genes that had a specific KEGG pathway annotation, 324 were down-regulated and were mainly associated with metabolism, growth, redox pathway, oxidative phosphorylation, the cell cycle, ubiquitin-mediated proteolysis, protein export and the MAPK (mitogen-activated protein kinases) signaling pathway.

**Conclusions:**

This work presents the first large scale gene expression study identifying the differences between schistosomula maintained in mice and those maintained in rats, and specifically highlights differential expression that may impact on the survival and development of the parasite within the definitive host. The research presented here provides valuable information for the better understanding of schistosome development and host-parasite interactions.

## Background

Schistosomiasis is one of the most widespread and prevalent parasitic diseases worldwide. More than 46 species of mammals have been reported to be naturally infected with *Schistosoma japonicum *(Chinese mainland strain) in China [1]. Two of the species are, mice and rats belong to the genera *Mus musculus *and *Rattus norvegicus*. Mice are permissive definitive hosts and support the full growth, development and sexual reproduction of the parasite. In contrast, rats are less susceptible since they do not provide a suitable micro-environment conducive for parasite growth and development [2]. The life cycle of *S. japonicum *in rat hosts is unsustainable, due to the low survival rate of cercariae penetrating through skin, compared to mice, and much fewer schistosomula successfully migrating from the liver portal circulation into the mesenteric veins, and finally in adult parasites a lower egg-laying rate and increased numbers of immature eggs [3]. Although the precise reasons for these features are unknown, previous investigations have indicated that the innate resistance in Wistar rats to *S. japonicum *might related to the presence of natural antibodies against the parasite (specifically immunoglobulin (Ig) G, G2a and G2c) and other humoral and/or cellular immune responses [4,5]. In a recent screen of an adult schistosome cDNA library [6], sera from Wistar rats as non-susceptible hosts were used to predict molecules involved in their resistance against *S. japonicum*.

In the present study, we have used microarray analysis to explore gene expression differences between schistosomula maintained in Wistar rats and those maintained in BALB/c mice, to enable the identification of parasite molecular mechanisms associated with the growth retardation of schistosomula in Wistar rats.

## Materials and methods

### Hosts and parasites

BALB/c mice (8 weeks, male, 20 g) and Wistar rats (8 weeks, male, 150 g) were purchased from the Shanghai Laboratory Animal Center, Chinese Academy of Sciences (Shanghai). New Zealand rabbits (male, 2.5-3.0 kg) were purchased from Feida Experimental Animal Co., Ltd. (Shanghai). The life cycle of *S. japonicum *(Chinese mainland strain from Anhui) was routinely maintained in New Zealand rabbits and *Oncomelania hupensis *(snail) in the Shanghai Veterinary Research Institute. For the experiment 45 Wistar rats and 45 BALB/c mice were subdivided into three groups of 15 each. Wistar rats, BALB/c mice and New Zealand rabbits were infected with 2000, 200 and 1500 cercariae, respectively. Infected animals were perfused using 37°C PBS at 10 days following infection and schistosomula collected. Parasites were extensively washed in 10 volumes of phosphate-buffered saline (PBS, pH 7.4). The study was approved (Project A001) by the Animal Ethics Committee of the Shanghai Veterinary Research Institute, Chinese Academy of Agricultural Sciences (CAAS) and the protocol approved by the Animal Care and Use Committee of the Shanghai Veterinary Research Institute, Chinese Academy of Agricultural Sciences (CAAS).

### Scanning Electron Microscopy observations

The length and width of individual schistosomula collected from Wistar rats and BALB/c mice (30 worms from each host species) were measured using light microscopy. Wistar rats and BALB/c mice (each group with 30 animals) were perfused to calculate the survival rate of *S. japonicum *worms. For SEM, schistosomula isolated from Wistar rats, BALB/c mice and New Zealand rabbits were fixed with 2.5% glutaraldehyde/PBS (pH 7.4) at 4-8°C, post-fixed with 1% (w/v) osmium tetroxide, and then with isopentyl acetate for 1.5 h before being dehydrated in an ascending ethanol dilution series. The samples were critical-point dried, and then coated with ~200Å of gold in an ion coater before SEM observations were performed using a JEOL6380LV microscope [7].

### Total RNA isolation, hybridization and feature extraction

Total RNA was isolated from schistosomula using TRIzol Reagent (Invitrogen, USA) and quantitated by ultraviolet spectrometry (Eppendorf Biophotometer) and using a Nano-Drop ND-1000 spectrophotometer (Thermo Scientific, USA). The quality of total RNA was assessed using a Bioanalyser RNA Pico Lab Chip (Agilent) before being stored at -80°C.

The microarray used in the study was constructed by Agilent Technologies (Santa Clara, USA) based on the transcriptomes of *S. japonicum*. The microarray design included 14,171 contiguous sequences (contigs) printed three times, plus proprietary positive and negative controls (Agilent Technologies). Contigs were based on the nucleotide sequences associated with a recent proteomics publication [8]. Microarrays were printed in a 4 × 44 k feature format. Full details of this schistosome microarray design have been deposited in the GEO public database (Gene Expression Omnibus, http://www.ncbi.nlm.nih.gov/geo/) with the associated platform (Accession no. GPL9759). The microarray data from the current study have also been deposited in GEO with the series accession no. GSE25728.

A 300 ng aliquot of total RNA was used to synthesize fluorophore-labeled cRNA using cyanine 3-CTP (CY3c) as described (Agilent Technologies: One-Color Microarray-Based Gene Expression Analysis). Samples were examined at A260 and A550 using a ND-1000 spectrophotometer to determine yield, concentration, amplification efficiency and abundance of CY3c. Microarray hybridizations were performed in duplicate for all samples.

### Data analysis, gene ontology and Kyoto Encyclopedia of Genes and Genomes (KEGG) pathway analyses

Microarray slides were scanned using an Agilent Microarray Scanner (B version) at 550 nm. The 'tag image format files' (tiffs) processed with the scanner were loaded into the Feature Extraction 9.5.3.1 image analysis program (Agilent) to produce standardized data for statistical analysis. All microarray slides were assessed for background evenness by viewing the tiff image by Feature Extraction. Feature-extracted data were analyzed using GENESPRING (version 7.3.1; Agilent Technologies/Silicon Genetics, Redwood City, USA). Microarray data were normalized using a normalization scenario for 'Agilent FE one-color', which including 'Data Transformation: Set measurements less than 5.0 to 5.0', 'Per Chip: Normalize to 50th percentile' and 'Per Gene: Normalize to median' [9,10].

Data sets were further analyzed using published procedures based on one-color experiments and ProcessedSignal values were determined using Feature Extraction software and GeneSpring microarray software (Agilent Technologies Version 7.3.1), including aspects of the signal:noise ratio, spot morphology and homogeneity. ProcessedSignal represents the signal after localized background subtraction and includes corrections for surface trends. Features were deemed 'Absent' when the processed signal intensity was less than twice the value of the processed signal error value. Features were deemed 'Marginal' when the measured intensity was at a saturated value or if there was a substantial amount of variation in the signal intensity within the pixels of a particular feature. Features that were neither absent nor marginal were deemed 'Present'. Data points were included only if they were present or present, absent, and probes or contigs were retained if all data points were present or present, absent [9,10].

Protein blast and GO analyses using Blast2Go Batch BlastX (six-frame translation protein homology) was performed at http://www.blast2go.de on all contigs. GO correlations with relative gene expression values were made using ErmineJ software [11]. In addition, the KEGG pathway of the differential expression genes was analyzed by using the maps available at http://www.genome.jp/kegg/[9,10].

### Validation of microarray data using qPCR analysis

A subset of six genes identified in schistosomula from Wistar rat as differentially expressed by microarray analysis, were validated using quantitative PCR (qPCR). Forward and reverse primers (Invitrogen, China) were designed according to the sequences of the eight contigs tested. Total RNAs of the schistosomula harvested from Wistar rats were isolated using TRIzol^® ^Reagent (Invitrogen, USA) following the manufacturer's instructions, quantified by ultraviolet spectrometry (Eppendorf Biophotometer), and then subjected to reverse transcription using a SuperScript™ III Reverse Transcriptase kit (Invitrogen, USA) and pd (N)6 random hexamer primers. The relative expression quality of schistosomula from Wistar rats was validated using the RG-3000A real-time PCR system (RoterGene, USA) and SYBR^® ^Premix Ex Taq™ (Perfect Real Time) kit (Takara, China). The qPCR reaction mixture (20 μl) contained 10-μl SYBR^® ^Premix Ex Taq™ (2×), 0.2 μl of the forward and reverse primers mixture, 1-μl cDNA template, and 7 μl RNase-free distilled H_2_O. Reaction conditions were as described in the SYBR green kit and the cycling protocol was as follows: 95°C for 10 s and 40 cycles of 95°C for 5 s, 55°C for 10 s and 72°C for 15 s, acquiring fluorescence at the end of each extension step; three repeats were carried out for each sample [12]. The PCR products were detected in real time using the Rotor-Gene 3000A Dual Channel Multiplexing System. The gene encoding NADH-ubiquinone reductase (Accession No. FN317713) was selected as a reference gene to normalize the expression differences in schistosomula from Wistar rats and from BALB/c mice. The primer sequences for the qPCR are listed in Table 1.

**Table 1:** Primers used for the validation of microarray data by quantitative qPCR analysis

Systematic Name	Protein Homology		sequences
Contig05321	Outer membrane protein	Forward	CAAGGTCCTGAAACGTGAAAC
		Reverse	GATGCTTCTACTTGCGTGTTAG
Contig03467	Arginine kinase	Forward	CGGTCGTCGTTTGTTTCTTC
		Reverse	AGAGTGCCACCCATGTTTG
Contig07888	Growth hormone-inducible transmembrane protein	Forward	GTGTGTCGATTAATGCTCAGTG
		Reverse	GGGTCCGCCAAGTAAACAG
Contig02569	Cell differentiation protein	Forward	GGGCGGAATAGAAGGAAACC
		Reverse	GCTTGGAGTATGACAGAGACG
Contig00624	14-3-3-like protein 2	Forward	CTTAACACCGAAGTCCAATGG
		Reverse	AACCAAGGCGAATAGGATGAG
Contig00468	Oxidoreductase HTATIP2	Forward	AAAGCCTTAATCAAAGCCCTTG
		Reverse	AGAGCACAGAAACCTACATCAG
Contig04397	NADH-ubiquinone reductase	Forward	CGAGGACCTAACAGCAGAGG
		Reverse	TCCGAACGAACTTT GAATCC

### Statistical analysis

Data are expressed as mean ± SD. Statistical analyses were performed by using the Student's t test. Values of p < 0.05 were considered to be significant. The analysis of correlation between microarray and quantitative PCR was performed in GraphPad Prism Version 5 (Graphpad Software Inc.) and was based on a previously published analysis [13].

## Results

### Morphological observations of schistosomula from Wistar rats

**Figure 1:**
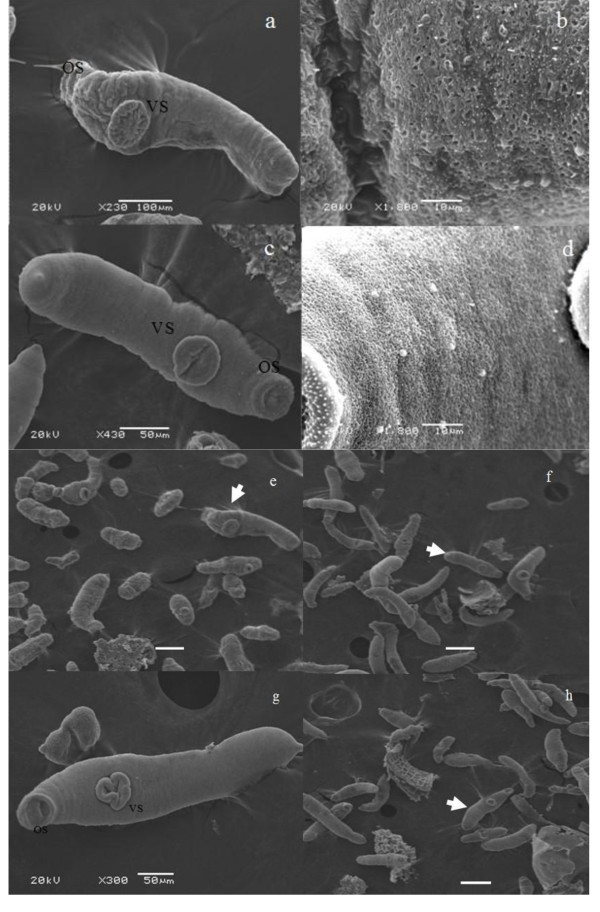
**Scanning electron microscopy**. Scanning electron microscopy (SEM) of schistosomula from Wistar Rat (a, b and e), from BALB/c mice (c, d and f) and rabbits (g and h): Os, oral sucker; Vs, ventral sucker. An overall wrinkled (a) and at higher magnification pitted (b) appearance is noted in schistosomula for Wistar rat hosts.(e, f and h Bar = 200 μm).

*S. japonicum *schistosomula were collected from Wistar rats 10 days post cercarial challenge. Comparison of the average length and width of schistosomula from BALB/c mice (length: 878.50 ± 137.45 μm; width: 159.10 ± 47.37 μm, n = 30), the length and width of schistosomula from Wistar rat were significantly (*p *< 0.01) reduced (828.3 ± 127.4 μm and 103.4 ± 22.8 μm, n = 30). Comparison of the survival rate of 10-day-old schistosomula maintained in BALB/c mice was ~ 70%, which was much higher than the rate of survival for the same age schistosomula maintained in Wistar rats at ~ 24%. Schistosomula maintained in Wistar rats compared to those maintained in BALB/c mice presented growth retardation and, when the surface topography was examined at the ultrastructural level, a withered, blebby appearance was evident (Figure 1). In general terms, the surface topography was similar in appearance to previous reports in *S. japonicum *lung schistosomula raised in mice [9]. The surface topography ultrastructure of schistosomula maintained in the two the permissive hosts, BALB/c mice and New Zealand rabbits, were similar (Figure 1 g and 1h).

### Microarray analysis of differentially expressed genes in schistosomula from Wistar rats and BALB/c mice

**Figure 2:**
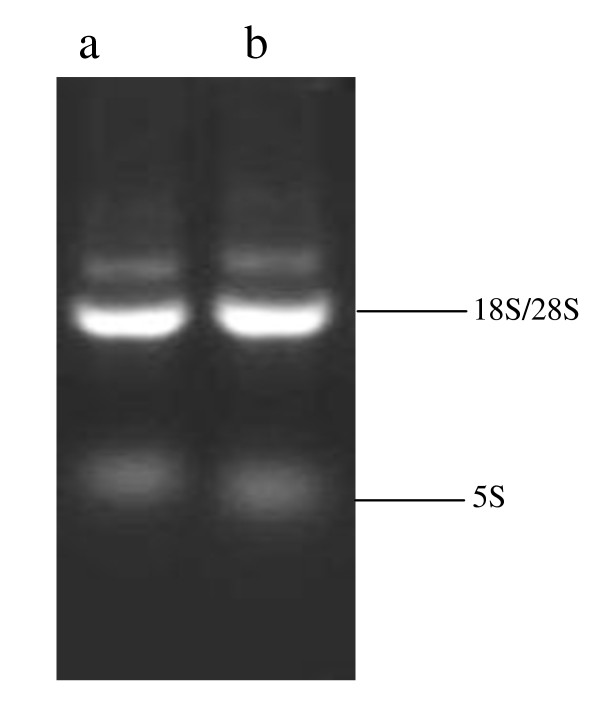
**Agarose gel electrophoretic analysis**. Agarose gel electrophoretic analysis of total RNA of the schistosomula from Wistar Rat and BALB/c mice (a): from Wistar Rat; (b): from BALB/c mice.

Total RNA was extracted from the schistosomula maintained in Wistar rats and BALB/c mice, and a prominent 18/28S ribosomal peak noted (Figure 2). Spectrophotometric and electrophoretic analysis showed the RNA to be of high purity and integrity and it was thus used for further study. The *S. japonicum *genome-wide oligonucleotide microarray was used to analyze the differences in gene expression between schistosomula maintained in Wistar rats and BALB/c mice. For genes to be considered differentially expressed, a two-fold or greater change in gene expression was required, with a t-test p-value ≤ 0.05.

**Figure 3:**
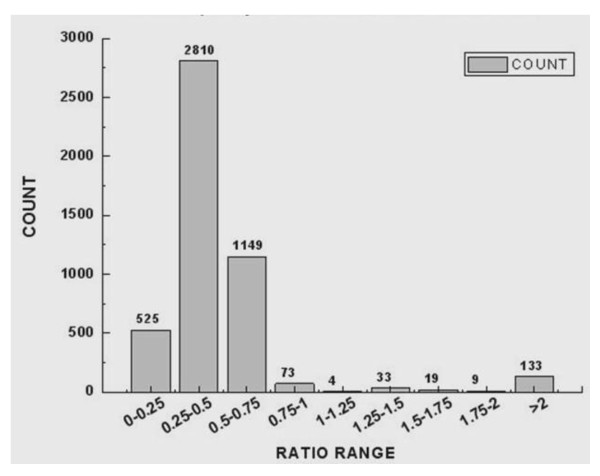
**The distribution of observed gene ratio**. The distribution of observed gene ratio values for the gene expression of the schistosomula from Wistar Rat compared with those from BALB/c mice. The majority of differential gene expression is seen in 2810 genes down regulated in parasites from Wistar rat host 0.25-0.5 ratio (2-4 fold down regulated).

**Figure 4:**
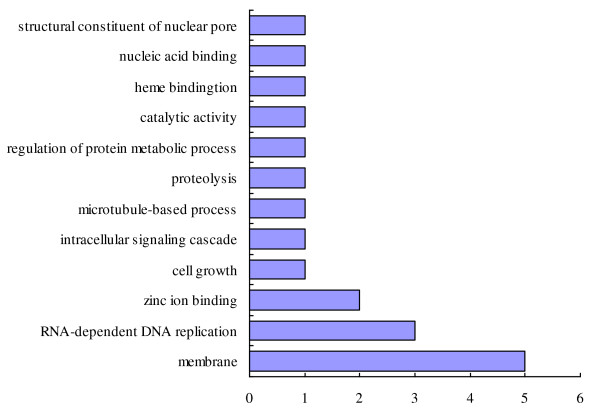
**Major gene ontology categories**. Major gene ontology categories for the up regulated genes (fold > 2) of schistosomula from Wistar Rat host. For each category the up regulated gene details are presented in Additional File 4.

**Figure 5:**
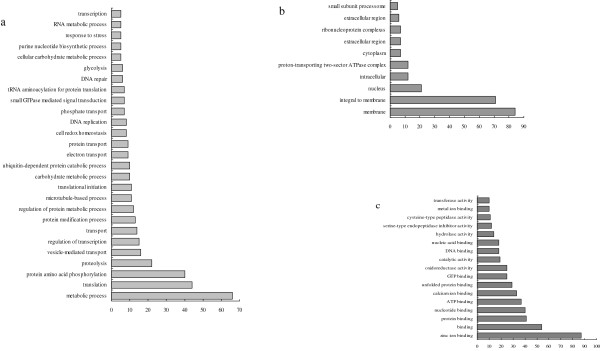
**Major gene ontology categories**. Major gene ontology categories for the down expressing genes (fold < 0.5) that were present in schistosomula from Wistar rat. **a. b. c**. represented the three main Go categories as biological process, cellular component, and molecular function. The category of the up regulated gene details in GO analysis was presented in Additional File 5.

All the gene expression data from the schistosomula from Wistar rats was normalized to those from BALB/c mice. Statistical analysis revealed a large number of differences in transcription in schistosomula maintained in the two hosts. The frequency distribution of the fold change of gene expression of schistosomula maintained in Wistar rats compared with those maintained in BALB/c mice shown in Figure 3. Of 3,468 genes, 3,335 were down-regulated (≤ 2 fold; Additional File 1) and 133 up-regulated (≥ 2 fold; Additional File 2) in schistosomula maintained in Wistar rats compared (normalized) with those maintained in BALB/c mice. Some differentially expressed genes of biological significance are shown in Tables 2 and 3. Of the 133 upregulated genes, 19 have assigned GOs; of these, 6 were regarded as having specific 'molecular functions', 5 were 'cell-component' specific and 8 were 'biological-process' specific involving 'binding', 'cell components', 'cellular processes' and other 'molecular functions' (Figure 4). Of the 3,335 down-regulated genes, 1,503 had GO assignments, of which 675 genes were predicted to have specific 'molecular functions'. These genes with molecular functions included 282 related to cell components and 546 were associated with particular biological processes. As shown in Figure 5, the GOs of the down-regulated genes included 'molecular', 'cellular component' and 'biological process' functions. Genes with 'molecular function' GO assignments were involved mainly in 'binding', 'oxidoreductase activity', 'catalytic activity', 'hydrolase activity', 'serine-type endopeptidase inhibitor activity' and 'cysteine-type peptidase activity'. Genes with 'biological process' assignments were involved mainly in 'metabolic processes', 'translation', 'protein and amino acid phosphorylation', 'proteolysis', 'vesicle-mediated transport' and 'regulation of transcription'. Those genes related to cellular components were mainly involved in 'membrane', 'membrane integrity', the' nucleus and intracellular components'.

**Table 2:** Examples of up regulated expressed genes in schistosomula from Wistar rat normalized with genes in those from BALB/c mouse

Systematic Name	Gene ID	Protein Homology	Fold Change
Contig03467	SJCHGC04932	Arginine kinase	2.05
Contig03162	SJCHGC04610	Regulating synaptic membrane exocytosis protein 2	2.28
Contig11651	SJCHGC13643	Tropomyosin	2.67
Contig11722	SJCHGC13718	Cyanobacterial phytochrome B	2.85
Contig10544	SJCHGC12455	Acetyl-CoA carboxylase 1	4.05
Contig01312	SJCHGC02645	Omega-3 fatty acid desaturase	4.76
Contig05321	SJCHGC06882	Outer membrane protein omp85	4.85
Contig00754	SJCHGC02003	Anaerobic nitric oxide reductase transcription regulator	4.98
Contig12392	SJCHGC14422	Cell surface receptor daf-4	5.03
Contig08614	SJCHGC10393	Splenin	5.30
Contig01305	SJCHGC02637	Dynein heavy chain	7.20
Contig02423	SJCHGC03839	Transforming growth factor beta-1	10.08
Contig11158	SJCHGC13108	Chaperone protein htpG	11.69
Contig00449	SJCHGC01393	Protein TAR1	27.13
Contig00716	SJCHGC01953	Pericentriolar material 1	60.36

A full list of genes, including systematic name, annotation microarray and protein homology, up regulated expressed genes in schistosomula from Wistar rat are presented in Supplementary Table 1. 2 Fold change refers to expression relative to those from BALB/c mice.

**Table 3:** Examples of down regulated expressed genes in schistosomula from Wistar rat normalized with genes in those from BALB/c mouse

Systematic Name	Gene ID	Protein Homology	Fold Ratio
Contig04100	SJCHGC05588	Methionine aminopeptidase 2	0.14
Contig06922	SJCHGC08562	Peptidyl-prolyl cis-trans isomerase	0.17
Contig05266	SJCHGC06822	Tegument antigen	0.20
Contig02700	SJCHGC04127	Arginine N-methyltransferase 2	0.23
Contig08320	SJCHGC10083	Thioredoxin peroxidase	0.23
Contig00624	SJCHGC01759	14-3-3-like protein 2	0.25
Contig06377	SJCHGC07996	Caspase-7	0.26
Contig00016	SJCHGC00098	Cathepsin S	0.26
Contig08226	SJCHGC09979	85 kDa calcium-independent phospholipase A2	0.30
Contig02277	SJCHGC03686	NF-kappa-B essential modulator	0.32
Contig01497	SJCHGC02852	Cathepsin B-like cysteine proteinase	0.33
Contig00468	SJCHGC01431	Oxidoreductase HTATIP2	0.35
Contig08025	SJCHGC09753	Glutathione S-transferase class-mu 28 kDa isozyme	0.40
Contig04318	SJCHGC05817	PDZ and LIM domain protein 2	0.40
Contig02569	SJCHGC03990	Cell differentiation protein RCD1 homolog	0.42
Contig00805	SJCHGC02061	23 kDa integral membrane protein	0.45
Contig00579	SJCHGC01663	Glycerol-3-phosphate dehydrogenase	0.45
Contig04815	SJCHGC06339	Antigen Sm21.7	0.46
Contig07888	SJCHGC09583	Growth hormone-inducible transmembrane protein	0.46
Contig00177	SJCHGC00800	Caspase-3	0.47

A full list of genes, including systematic name, annotation microarray and protein homology, up regulated expressed genes in schistosomula from Wistar rat are presented in Additional File 2. 0.5 Fold change refers to expression relative to those from BALB/c mice.

Examining all of the differentially expressed genes, 328 had a specific KEGG pathway annotation; of these, 324 were down-regulated and were mainly involved in 'oxidative phosphorylation', 'glycolysis' and/or 'gluconeogenesis', the 'citrate cycle' (TCA cycle), 'metabolism', the 'cell cycle', 'ubiquitin-mediated proteolysis', 'aminoacyl-tRNA biosynthesis', 'protein export', '*N*-glycan biosynthesis', 'basal transcription factors' and the 'MAPK (mitogen-activated protein kinases) signaling pathway' (Additional File 3).

### Analysis of development, growth, tegument formation and redox pathway-associated genes

Further analyses of differentially expressed genes revealed that some are involved in the development of the MAPK (mitogen-activated protein kinases) cellular signaling pathways, such as mitogen-activated protein kinases (contig01000, contig04011, contig04351 and contig04389) and mitogen-activated protein kinase scaffold protein (contig04351), all of which were significantly down-regulated in schistosomula maintained in Wistar rats. Some genes encoding growth-related factors, such as growth factor receptor-bound protein 2 (Contig03417) and growth hormone-inducible transmembrane protein (Contig07888), were also down- regulated. Various membrane-related genes (tetraspanin-3 (Contig04646), tetraspanin-4 (Contig07472), tetraspanin-11 (Contig04240) and tetraspanin-18 (Contig04880) were also down-regulated, which suggests that the developmental disfunction ariseing from the down- regulation of these genes in schistosomula maintained in the rat host might partially contribute to the observed abnormal tegument architecture. Proteins in the cell redox pathway were also noted as down-regulated including thioredoxin peroxidase (Contig08320, Contig11578), thioredoxin H-type 2 (Contig08215), thioredoxin-like protein (Contig00886, Contig04053) and thioredoxin domain-containing proteins (Contig01730).

### qPCR validation

**Figure 6:**
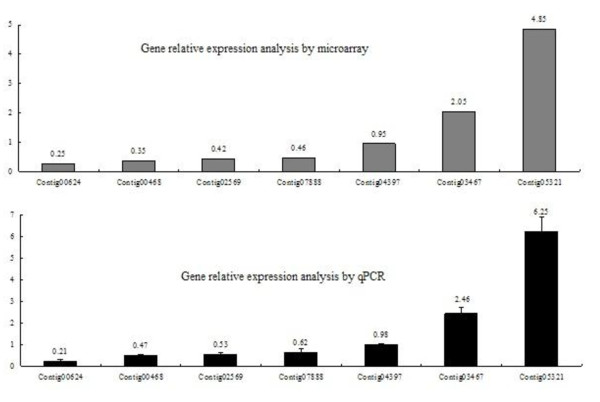
**Result of qPCR confirmation of gene microarray data subset**.

Six genes with varying biological functions were selected for qPCR so as to validate the microarray transcription results. The primer sequences for the qPCR are listed in Table 3. NADH-ubiquinone reductase was used to normalize any loading differences.. Additionally, the expression of the six selected genes was normalized by the expression of those from BALB/c mice (the relative gene expression in those from BALB/c mice were used to standardize the ratio) [11]. As shown in Figure 6, the results of the microarrays and qPCR closely associated with each other.

## Discussion

It has been noted that *S. japonicum *as a zoonosis can infect more than 40 species of mammals including cattle, sheep, goat, rabbit and mice, all of which are susceptible to the infection, while species such as water buffalo, pig and rat, are less susceptible as indicated by low parasite developmental rate and a smaller worm size in these hosts [1]. Research from our group has investigating stage-specific genes and different host- sourced genes. This work has helped us to identify key molecules associated with parasite development and survival, and provided insight into the interplay between *S. japonicum *and its definitive hosts [10,14].

Previous research by others has shown that susceptibility to *S. japonicum *varies among different rodents hosts in endemic areas, and the spectrum of more to less susceptible species included: *Mus musculus (Apodemus agrarius), Rattus eloquens, R. hainanicus, R. flavipectus, R. rattoides , R. norvegicus and Microtus fortis *[1]. When Chinese field rats (*R. norvegicus*) were infected with *S. japonicum *under laboratory conditions, only 12-30% of the initial cercarial challenge were later found five weeks later as adults in the portal mesenteric veins, and in addition the size of both male and female worms were small and no eggs were found in the feces during the subsequent six-month post-infection period [15]. This contrasts with most mouse strains which appear to be permissive hosts, with the exception of one strain (129/J) which has been shown to be relatively resistant as reflected by low maturation rates of day 40 adult worm numbers [16]. Based on our research, an approach to understand schistosome - definitive host interplay is to focus on parasite defensive mechanisms and host reactive responses [17,18].

The morphology of schistosomula collected from Wistar rats observed using SEM demonstrated obvious differences when compared to those from BALB/c mice. The former are characterized by a withered, blebbed appearance and an irregular body surface. Similar morphological changes to the schistosome tegument have been reported due to host immunological damage of adult parasites in Sj26-GST immunization in BALB/c H-2b mice (Chinese mainland strain, Anhui isolate) [19]. The current study also showed that schistosomula from Wistar rats present retarded growth as well as a dramatic reduction of the associated pathological impact on the host. The developmental maturation rate of *S. japonicum *in BALB/c mice is approximately 70% [17]; however this is much less in parasites from Wistar rats. All of the data presented here confirms that the growth and the development of schistosomes are delayed or arrested in this rat strain.

The function of most of the schistosomula genes identified in this study as differentially modulated have not be elucidated. However, from the better annotated genes we can obtain some insights in the impact of the host on the developing parasite. Genes encoding pericentriolar material 1 protein (PCM1) and tropomyosin were two of the most highly expressed genes identified in the schistosomula maintained in Wistar rats. PCM1 is a key molecule in the biogenesis, function and maintenance of centrosomes and cilia in animal cells. PCM1 also acts as a scaffold for several proteins which are then transported to the centrosome in a microtubule-dependent manner via dynein-dynactin molecular motors [20]. PCM1 is also thought to be involved in recruiting proteins necessary for centrosome replication and in organizing or anchoring microtubules emanating from the microtubule-organizing center (MTOC) [21]. The exact function of PCM1 is unknown and further investigation of this molecule in the worms obtained from Wistar rats would be interesting and may represent increased levels of cell division. Tropomyosin is a major regulatory molecule of the contractile system in muscle as well as an important cytoskeletal component of non-muscle cells, and has been considered as a schistosome vaccine candidate [22]. Our current report of the over expression of tropomyosin in the schistosomula maintained in Wistar rats might indicate the stimulation of a strong immune response against the parasite, leading to poor growth of the worms.

The GO and KEGG pathway analysis of down-regulated genes in schistosomula from Wistar rats were associated predominantly with important biological process, including 'metabolic processes', 'growth', 'redox pathways', 'membrane and MAPK signaling pathway' molecules. These molecular functions are highly likely to be associated with the development and growth of the schistosome [23]. *S. japonicum *parasites are unable to synthesize some key nutrient molecules, such as fatty acids, sterols, purines, nine essential amino acids, arginine and tyrosine [8]. The results of the current study demonstrate that the metabolic formation of several nutrients, as reflected in transcriptional down regulation, occur in schistosomula maintained in Wistar rats> These includes genes related to the synthesis of arginine, proline, glycine, serine, threonine, fatty acids and vitamin B6, suggesting that the metabolism status of the parasite within a rat host, is possibly one of the key reasons for the growth suppression of worms.

Peroxiredoxins are a large family of peroxidase genes that have important antioxidant and cell-signaling functions [24]. One of the established survival mechanisms for schistosomes in the definitive host is the production of protective antioxidant proteins which act to neutralize oxidative damage resulting from the host immune response, as well as damage from parasite self-generated oxygen radicals [25,26]. As shown in the results presented here, some genes involved in the redox pathway, such as those encoding thioredoxin peroxidase and other thioredoxin domain-containing proteins, were down-regulated in worms maintained in the rat host. In addition, it has been reported that levels of H_2_O_2 _and O^2- ^in different mammalian species differs and the level of these reactive oxygen species (ROS) in Wistar rats was higher than those in the mice [27]. Reviewing these two observations, we suggest that the high levels of host ROS in Wistar rats may impact on parasite damage and parasite ROS-related enzyme activity, as reflected in the gene expression in the schistosomula.

Several parasite genes involved in a range of signal pathways have been identified as key factors contributing to the development and growth of schistosomes. These including genes encoding Wnt, notch, insulin receptor hedgehog, MAPK and transforming growth factor β (TGF-β); all of which are growth factors, receptors and essential components which regulate many cellular processes during organogenesis and tissue development [28,29]. The data presented in our study shows that multiple genes related to development and growth processes, such as those within the MAPK gene family and growth factor receptor-bound proteins, were significantly down-regulated in parasite maintained in rats, and are likely to impact on observed lack of growth in these worms.

## Conclusions

In conclusion, the current study has shown that schistosomula maintained in Wistar rats compared to parasites maintained in BALB/c mice, demonstrate morphological damage to the parasite surface, growth retardation and pathological change to the host. These phenotypes may be associated with distinctive differential expression associated with development, growth, metabolism, redox and signal transduction-related genes. Many of these modulated parasite genes are significantly influenced by the local environment during their development with the definitive host; and in turn impacts on parasite survival and subsequent development. These factors ultimately lead to the growth disruption of schistosomula in the unsusceptible Wistar rat host. These parasite genes appear central for the survival, growth and development of the schistosome in their final hosts, and could be potential targets for vaccine candidates or new drugs for the control of schistosomiasis in the future.

## Competing interests

The authors declare that they have no competing interests.

## Authors' contributions

JL, JP Conceived and designed the experiments; JP, GNG, HH, YH, WJ, XW, ZF, JL and YS performed the experiments and analysed the data. All authors read and approved the final manuscript.

## Supporting information

Table S1 The full list of down regulated genes, including systematic name, Gene ID and protein homology and ratio, Differentially expressed gene in schistosomula from Wistar Rat of 0.5 ratio refers to 2 fold expression relative to those of schistosomula from BALB/c mice.

Table S2 A full list of genes, including systematic name, Gene ID and protein homology, up regulated in schistosomula from Wistar Rat 2 Fold change refers to expression relative to those of schistosomula from BALB/c mice.

## Supplementary Material

Additional file 1 **The full list of down regulated genes, including systematic name, Gene ID and protein homology and ratio, Differentially expressed gene in schistosomula from Wistar Rat of 0.5 ratio refers to 2 fold expression relative to those of schistosomula from BALB/c mice**. Click here for file

Additional file 2 **A full list of genes, including systematic name, Gene ID and protein homology, up regulated in schistosomula from Wistar Rat 2 Fold change refers to expression relative to those of schistosomula from BALB/c mice**. Click here for file

Additional file 3 **KEGG analysis of differential expression genes in the schistosomula from Wistar Rat compared with those from BALB/c mice**. Click here for file

Additional file 4 **The category of the down regulated gene details in Gene Ontology analysis**. Click here for file

Additional file 5 **The category of the up regulated gene details in Gene Ontology analysis**. Click here for file
